# Characterizing the US trade in lionfishes

**DOI:** 10.1371/journal.pone.0221272

**Published:** 2019-08-15

**Authors:** Timothy J. Lyons, Quenton M. Tuckett, Jeffrey E. Hill

**Affiliations:** University of Florida/IFAS, SFRC Program in Fisheries and Aquatic Sciences, Tropical Aquaculture Laboratory, Ruskin, FL, United States of America; University of Plymouth, UNITED KINGDOM

## Abstract

Invasive lionfishes *Pterois volitans* and *Pterois miles* have spread throughout the tropical western Atlantic Ocean, Gulf of Mexico, and Greater Caribbean. Beyond these two invaders, additional species within the subfamily Pteroinae are regularly imported into the United States. We evaluated the trade of lionfishes as a surrogate measure for propagule pressure, an important component of invasion success. Proactive evaluation of marine ornamental fishes in trade is vital, particularly for those sharing characteristics with known invaders. We utilized one year of import records from the U.S. Fish and Wildlife Service’s Law Enforcement Management Information System database and two domestic databases to capture the trade of all lionfishes in the US, the invasive complex in its invaded range in Florida, and two Hawaiian endemic lionfishes. Retail surveys were completed to assess lionfish availability across 10 coastal states. Compared to species diversity within the subfamily, the number of traded species was low and just two species were traded at moderate to high volume, including *P*. *volitans* and *Dendrochirus zebra*. At the retail level, fewer species are available to consumers. The trade in lionfishes is consolidated because most lionfishes originate from two Indo-Pacific countries and arrive through the port of Los Angeles. The volume and diversity of traded lionfishes presents some risk of introduction for lionfishes which are not established, and secondary introductions of the invasive *P*. *volitans*. In combination with rapid risk screening, this research can be applied to a proactive risk management framework to identify risky species prior to introduction and establishment.

## Introduction

**Fig 1:**
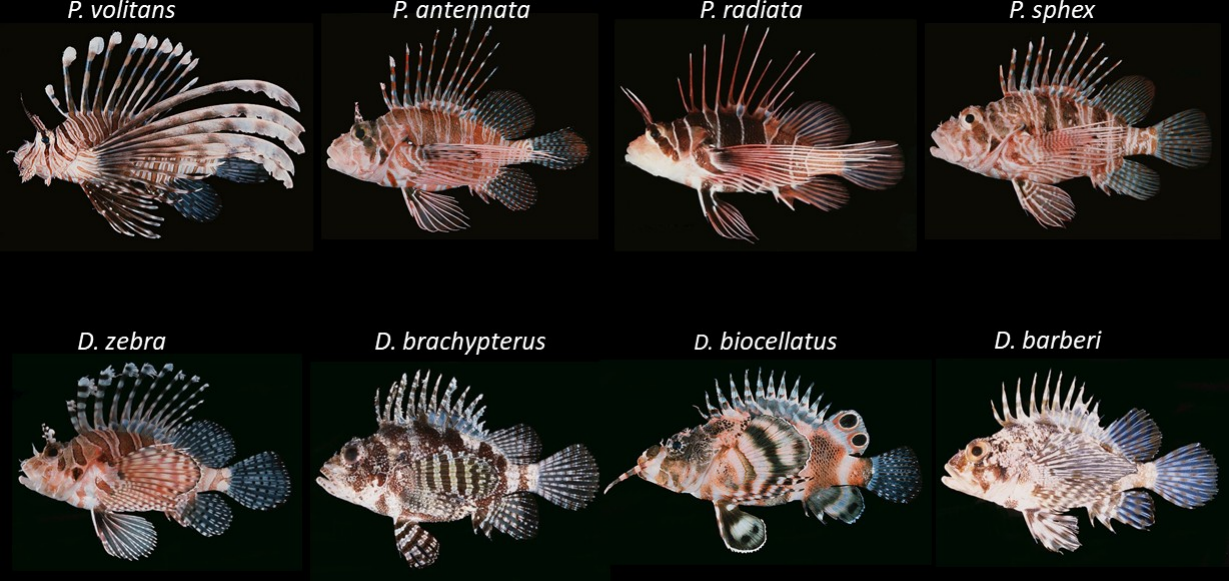
Eight species of lionfishes present in the U.S. ornamental fish trade from April 2016–2017. Genera *Pterois* (top) and *Dendrochirus* (bottom). Images collated with express permission from: Randall, J.E., 1997. Randall's tank photos.

The well-developed global trade in marine ornamental species supports collectors, wholesalers, and retailers economically, and can produce conservation benefits through public exposure and outreach [1]. However, the global trade in these species is not without its drawbacks, including the potential introduction and establishment of non-native species [2], which can lead to economic, social, and ecological costs [3–5]. Eradicating or slowing the spread of introduced species post-establishment can be extremely difficult [6]. Environmental damages and control programs for invasive marine and freshwater fishes in the U.S. cost managers and stakeholders an estimated US$5.4 billion each year [3]. The global trade in marine ornamental species encompasses over 1800 species of fishes from at least 125 different families, including taxa from the small-bodied *Chromis viridis* (10.0 cm) to the largest member of the family Labridae, *Cheilinus undulates* (229.0 cm) [7–8]. Included in trade are the ecologically and economically important venomous lionfishes in the subfamily Pteroinae (Fig 1), a group with known invaders. As such, it is important to evaluate the variety and volume of potentially risky species in the marine ornamental trade to inform proactive management approaches.

A prominent example of a marine invasion of globally traded aquarium species is the invasive lionfish complex that includes *Pterois volitans* and *P*. *miles* which have established and spread throughout the tropical western Atlantic Ocean, Gulf of Mexico, and Caribbean [9]. *Pterois miles* is also spreading quickly through the Mediterranean as a Lessepsian migrant through the Suez Canal [10–12], further highlighting the invasion potential of the subfamily. These two widespread invaders have documented impacts on ecosystem structure and function throughout their invaded range [13–16]. Because invasion history and propagule pressure have a strong influence on the likelihood of establishment [17], considering the large number of species in the global aquarium trade [8], and acknowledging that resources for risk assessment and invasive species management are highly limited, an appropriate proactive management approach would focus first on evaluating risk for groups such as the Pteroinae which have members with a history of invasion and associated economic, social, and ecological costs.

Propagule pressure is often directly related to establishment probability [17–20] and thus is an important component of current risk assessment methods [21–23]. Spatial and temporal distribution, as well as the number and frequency of propagules, greatly influences the ability of an invader to overcome environmental and demographic stochasticity and ultimately establish [19,24–25]. Although propagule pressure is an important predictor of establishment, it is difficult to measure directly because data associated with the early stages of invasion and failed invasions are often absent [18]. As such, researchers utilize surrogate measures to indirectly estimate propagule pressure, such as the movement of visitors within nature reserves [26], shipping and boating traffic [27], or the movement of live marine fishes in the ornamental aquarium trade [28].

Here we characterize the ornamental trade pathway for the subfamily Pteroinae in the United States. Our goal was to identify the taxonomic composition and volume of traded lionfishes, their collection origin, major receiving ports, and occurrence in retail outlets to inform proactive risk management. Importation was investigated using import records from the U.S. Fish and Wildlife Service’s Law Enforcement Management Information System (LEMIS) database, a central repository used to record wildlife arriving in the United States. These data were supplemented by two domestic databases to capture the collection and trade of the invasive complex from its invaded range, and two species of lionfishes endemic to Hawaii, which are not reported under the LEMIS system. *Pterois volitans* was once the 29^th^ most frequently traded marine fish by volume [8], which may have contributed to elevated propagule pressure and thus a greater chance for establishment. However, the trade volume and species composition of other lionfishes in the genera *Pterois*, *Dendrochirus*, *Parapterois*, *Brachypterois*, and *Ebosia* have not been evaluated in detail. The retail-level trade in lionfishes, the level directly suppling lionfishes to hobbyists, has not been investigated. Therefore, we conducted a survey of lionfish availability in retail aquarium stores within ten coastal states with access to potentially suitable marine habitat, as a comparative measure to import records. This information on potential invasion pathways can be especially useful when paired with rapid risk screening protocols to identify risky species that are present in high volume.

## Methods

While efforts are underway to increase the number of marine species in captive production [29], the trade in marine ornamental fishes is supplied primarily by the capture and transport of wild organisms [7]. To date, there are no reports of captive culture for any species in the subfamily Pteroinae. As such, all specimens are collected from their native or introduced ranges. All lionfishes are shipped to the United States via air transport. The trade pathway from collector to hobbyist is characterized by a complex chain of custody that presents some challenges to traceability and monitoring efforts [30], but typically includes consolidation at foreign export facilities, departure from foreign exporter, arrival at domestic importer, distribution from importer to wholesaler, and distribution from wholesaler to retailer [30–31].The escape or intentional release of specimens during transport and at points of consolidation is unlikely because of packaging and shipping practices and standards [31]. The risk of escape or release is highly concentrated at the end user of the pathway, at the hobbyist level [31].

### Data collection

This research was granted a formal exemption waiver under University of Florida #IRB201900167. The LEMIS database was accessed through a Freedom of Information Act (FOIA) request for data from April 2, 2016 through April 1, 2017 (S1 Dataset). The LEMIS database includes electronically submitted and manually entered USFWS 3–177 forms (Declaration for Importation or Exportation of Fish or Wildlife) required by the Code of Federal Regulations (CFR) title 50 part 14 [32]. Relevant fields in the requested Standard Declaration Report include identification of imported lionfishes to the species level, quantities of imported lionfishes, the foreign country of origin, the foreign country of export, and the domestic receiving port. Additional proprietary information is collected, but redacted prior to fulfilling a FOIA request. Only records with the wildlife designation indicating that the shipment was comprised of live individuals (LIV) were included in the analysis. An additional 3,351 lionfish were excluded from the analysis because they were imported as preserved specimens (SPE) or jewelry (JWL).

Because the LEMIS database applies only to trade originating from outside the United States, it does not report domestic collection or transport of lionfishes. We included the collection and trade of the invasive complex *P*. *volitans*/*P*.*miles* in Florida (the primary collection site for the invasive population) using the Florida Fish and Wildlife Conservation Commission Fish and Wildlife Research Institute’s Annual Commercial Fishery Landings database (S2 Dataset). This database includes the Florida county of collection, the quantity of lionfish collected, the number of commercial trips taken, and the value of collected lionfish. Reporting is gathered from trip-ticket requirements for commercial landings [33]. The volume of *P*. *volitans* sourced from adjacent U.S. states is thought to be negligible but may increase these numbers slightly. Additionally, we included the trade of two Hawaiian species *Pterois sphex* and *Dendrochirus barberi* by submittal of a Request for Commercial Fishing Report Information to the Hawaiian Division of Aquatic Resources (DAR). This database includes time of harvest, quantity, and value of lionfishes collected from commercial fishing [34].

### Retail surveys

The occurrence of lionfishes in 168 retail stores within 10 coastal U.S. states was evaluated during a two-week period from June 29^th^ to July 12^th^, 2017 (S3 Dataset). States were selected to reflect the distribution of the invasive lionfish complex in US waters and thus regions where warm climatic conditions are most likely to support the survival of other species of lionfishes [35]. California was included because *P*. *volitans* has a thermal niche that may allow for permanent established populations in some areas of southern California [36], and because it is a major hub for the marine ornamental fish trade [37]. Retail stores were identified and selected using a standardized Google search for the term “saltwater aquarium store in” followed by the state where that store is located. To meet selection criteria, identified retailers had to 1) sell live saltwater fishes, 2) maintain regular business hours (i.e., stores by appointment only were excluded), 3) sell directly to the public, and 4) have a listed phone number. Retail stores were selected in the order that they appeared in the search. The number of retail stores surveyed in each state was determined by that state’s population reported by the 2010 United States Census Bureau to reflect the positive relationship between population size and the potential for introduction [38–39]. California was assigned an arbitrary value of 50 representative stores because it has the largest population of any U.S. state. Texas (34), Florida (26), Georgia (14), North Carolina (12), Virginia (10), South Carolina (6), Alabama (6), Louisiana (6), and Mississippi (4) were assigned a representative number of retail stores proportional to state population.

The survey used a standardized script format, in which the surveyor identified themselves, the intent and purpose of the study, asked about species availability for all species in the subfamily Pteroinae, and included the option to opt out of the survey. Common names were verified with purchasing lists when available. Each available species was recorded as a unique hit, where multiple individuals of the same species at one location resulted in just one hit for that species. Stores unwilling to disclose stock lists were recorded as “non-participant.” Stores with nonfunctional listed telephone numbers, or those that did not answer the store’s listed telephone after three attempts were recorded as not available (N/A). Both non-participant and N/A occurrences were included in the total number of stores surveyed but were excluded from percent occurrence to reflect uncertainty in species availability.

## Results

**Fig 2:**
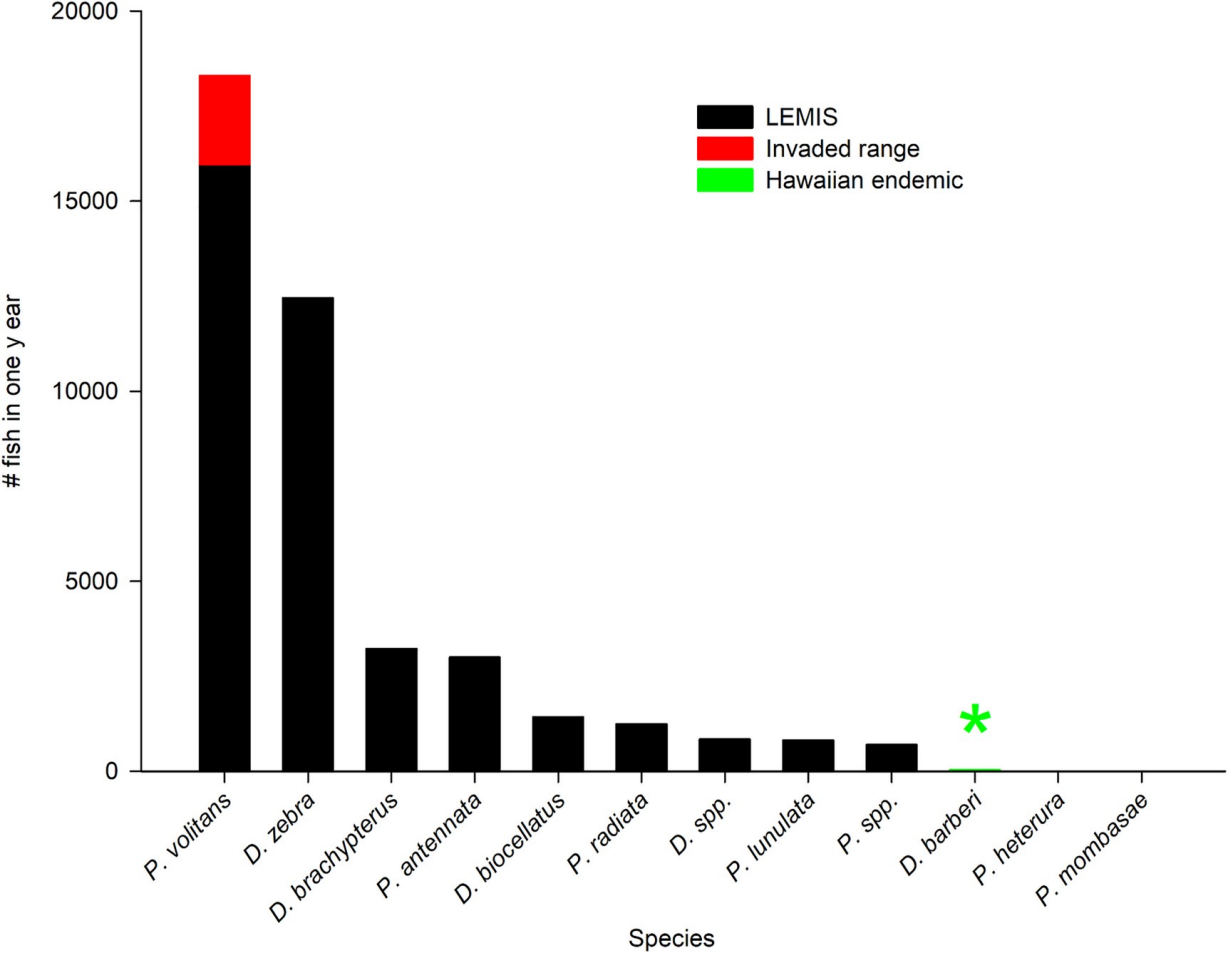
Total volume of lionfish species imported into the United States from April 2016–2017. Black bars indicate records in the U.S. Fish and Wildlife Service’s LEMIS database. Red bar indicates *P*. *volitans* collected in Florida (invaded range). Green arrow indicates *P*. *sphex* and *D*. *barberi* collected from the Hawaiian Islands. Reported volume of *D*. *barberi* = 32, *P*. *heterura* = 11, *P*. *mombasae* = 2. The quantity of *P*. *sphex* was unavailable because a single commercial collector reported landings, therefore trade volume was deemed proprietary by the Hawaiian Division of Aquatic Resources.

Between April 2016 and April 2017, 39,648 live Pteroinae of 9 species were imported into the United States. An additional 2,329 lionfish individuals were collected from Florida and 32 individuals were collected from Hawaii (Fig 2). Overall, 57.2% of live imports were in the genus *Pterois*, 42.7% in the genus *Dendrochirus*, and just 0.03% in the genus *Parapterois*. The genera *Brachypterois* and *Ebosia* were absent from the data sources. Of the 21,711 imported *Pterois*, 3.2% were not identified to the species level. Of the 17,926 imported *Dendrochirus*, 4.8% were not identified to the species level. *Pterois volitans* accounted for 40.2% of all lionfishes imported, followed by *Dendrochirus zebra* which accounted for 31.5% of imports (Fig 2). Five species *Dendrochirus brachypterus*, *Pterois antennata*, *Dendrochirus biocellatus*, *Pterois radiata*, and *Pterois lunulata* were traded in comparatively moderate to low volumes, and three species were traded in very low volumes, including *D*. *barberi* with just 32 individuals, *Parapterois heterura* with 11 individuals, and *Pterois mombasae* with 2 individuals (Fig 2). Hawaiian export volume for *P*. *sphex* was unavailable because only a single collector targeted that species from April 2016 to April 2017 and therefore the data were redacted as proprietary.

**Fig 3:**
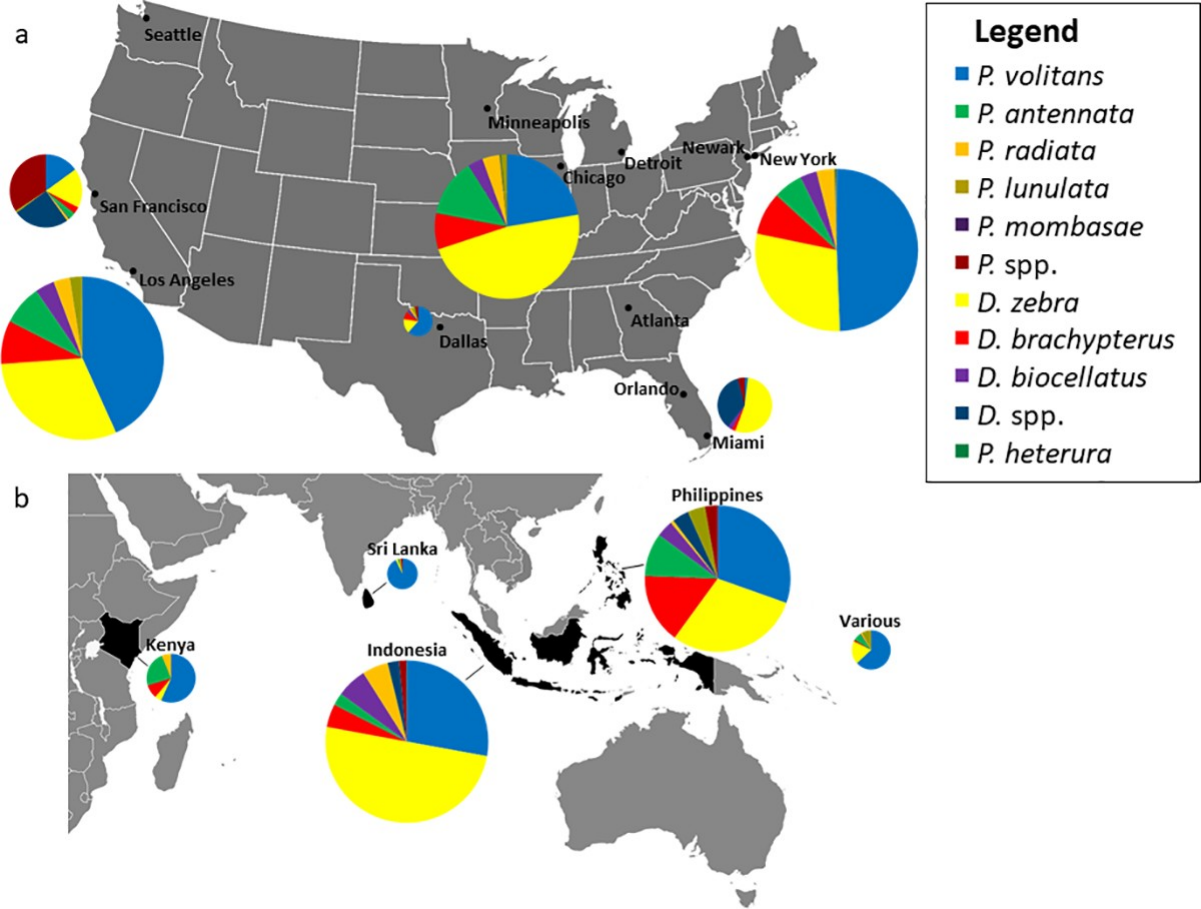
Species composition and volume of lionfish trade for six major receiving ports and five major countries of origin. (a) Major receiving ports include Los Angeles, New York, Chicago, San Francisco, Miami, and Dallas/Ft. Worth) and accounted for 98.6% of all lionfish imports into the United States from April 1, 2017–2018. Size of pie chart is proportional to total trade volume received by that port. The port of Los Angeles is reduced in scale by 856% to equal the area of the second largest port and accounts for 74.0% of all lionfish imports (n = 29,414). Ports that received lionfishes, but did not comprise more than 1% of total trade volume are included as additional named cities. Spp. represents fishes that were not identified to the species level. (b) Major collection origins include Indonesia, Philippines, Kenya, various, and Sri Lanka and accounted for 99.2% of all collections. “Various” represents fishes that were sourced from multiple countries.

**Fig 4:**
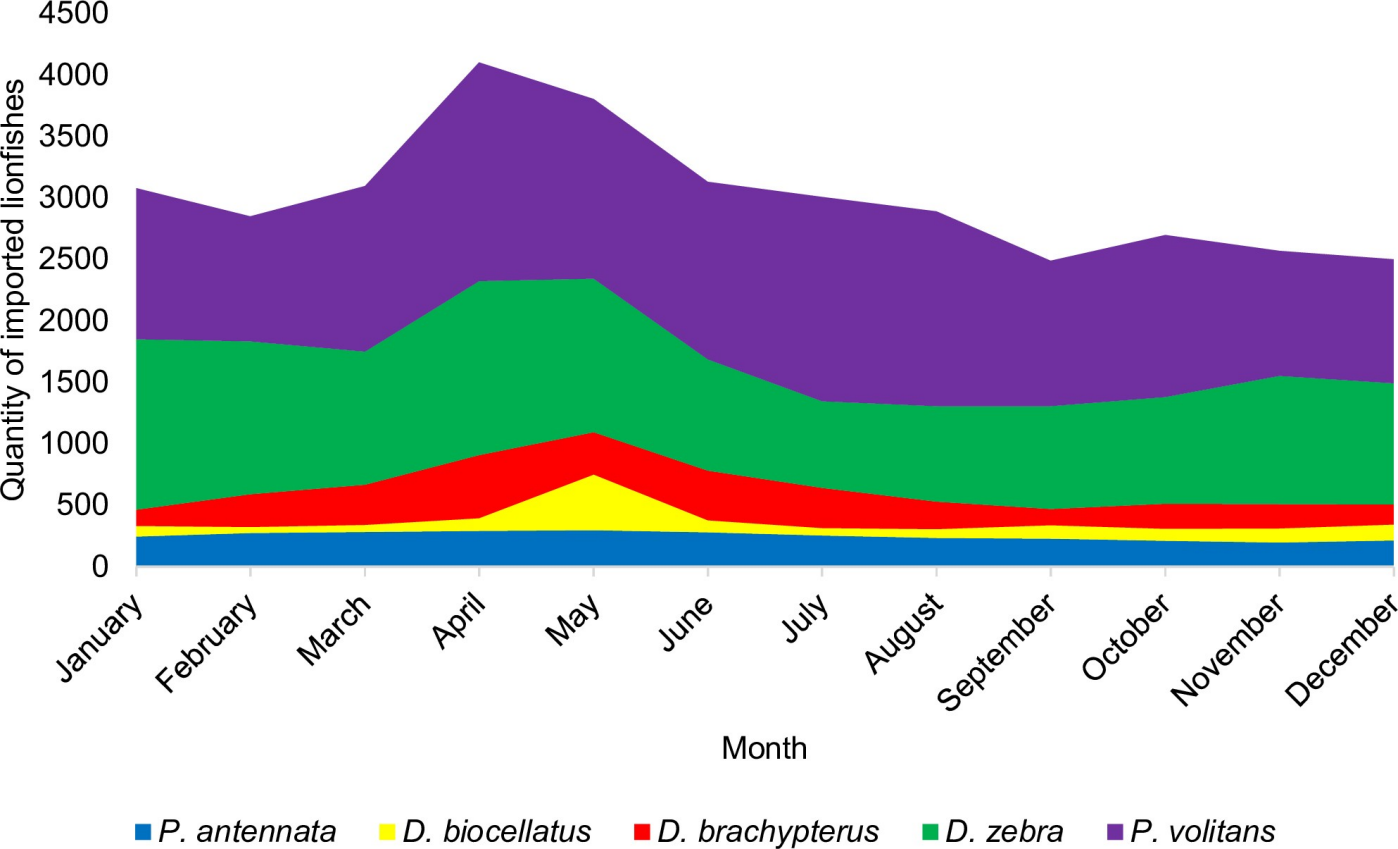
Monthly variability for the top five species of lionfishes by import volume from April 2, 2016 to April 1, 2017.

Four countries accounted for 90.3% of the total number of lionfishes collected including, in order of total quantity, Indonesia, the Philippines, Kenya, and Sri Lanka (Fig 3B). An additional 9.1% of lionfishes were listed under the origin designation various (VS), which denotes shipments of lionfishes sourced from multiple countries. Two countries, Indonesia and the Philippines, accounted for 71.8% of live lionfish imports (Fig 3B). The collection of *P*. *volitans* in Florida comprised only 12.3% of total trade volume for this species (Fig 2). There were few noticeable trends in the seasonal availability of lionfishes by species, but overall lionfish imports were highest in April and May (Fig 4).

**Fig 5:**
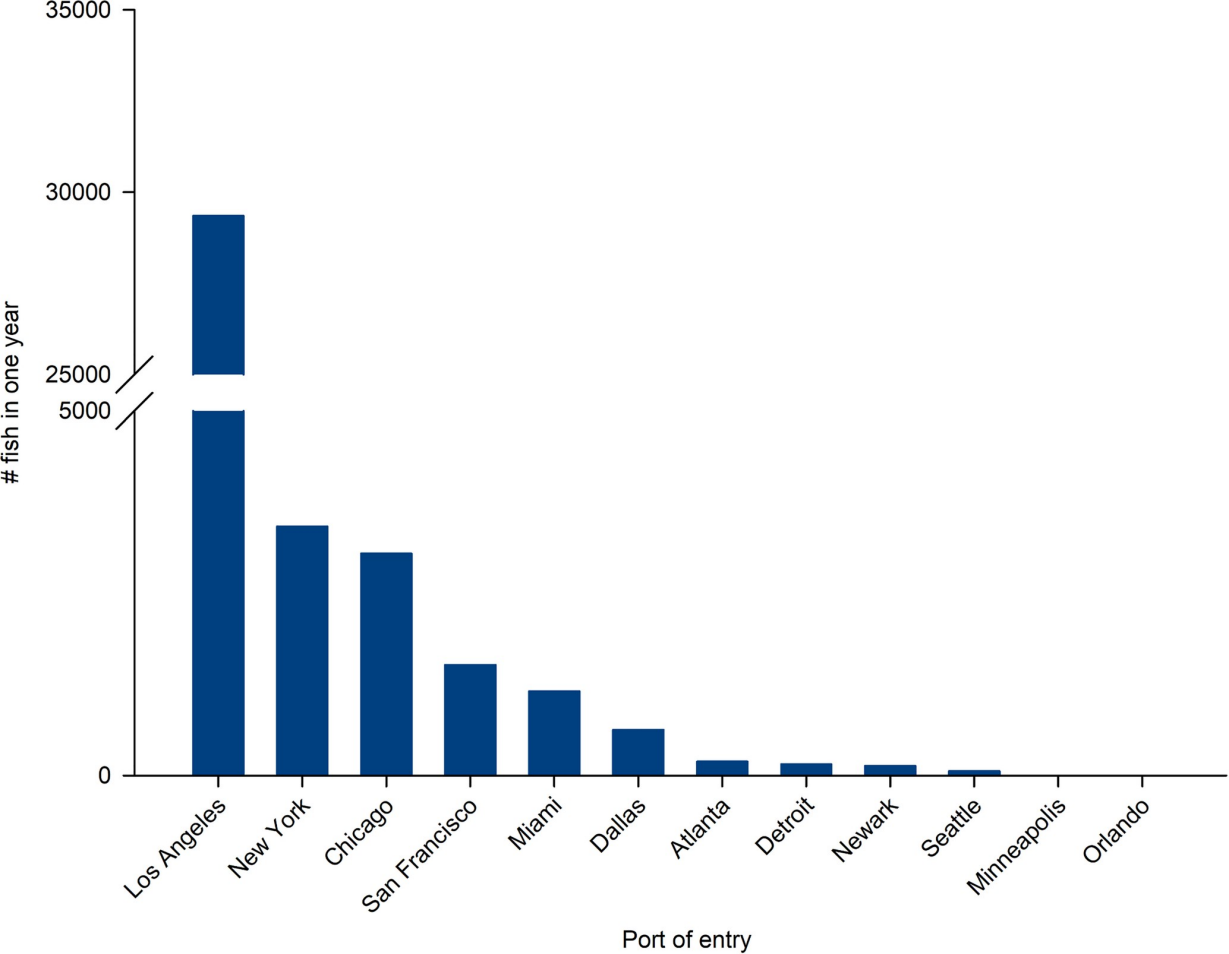
Total volume of lionfish received at ports in the United States between April 2, 2016-April 1, 2017. Values reported from the U.S. Fish and Wildlife Service LEMIS database (n = 39,648). Los Angeles = 74% of imported lionfish; New York = 8.6%; Chicago = 7.7%; San Francisco = 3.8%; Miami = 2.9%; Sum of all other ports = 2.9%.

The port of Los Angeles received 74% of the lionfishes imported into the U.S., followed by New York (8.6%), Chicago (7.7%), San Francisco (3.8%), and Miami (2.9%) (Figs 3A and 5). Additional receiving ports included Dallas, Atlanta, Detroit, Newark, Seattle, Minneapolis, and Orlando, but these ports in aggregate accounted for only 2.9% of total imports (Fig 5). Of 1,156 lionfishes received by the port of Miami, nearly 40% were not identified to the species level. On average, individual shipments included an average of 8.2 individuals.

**Fig 6:**
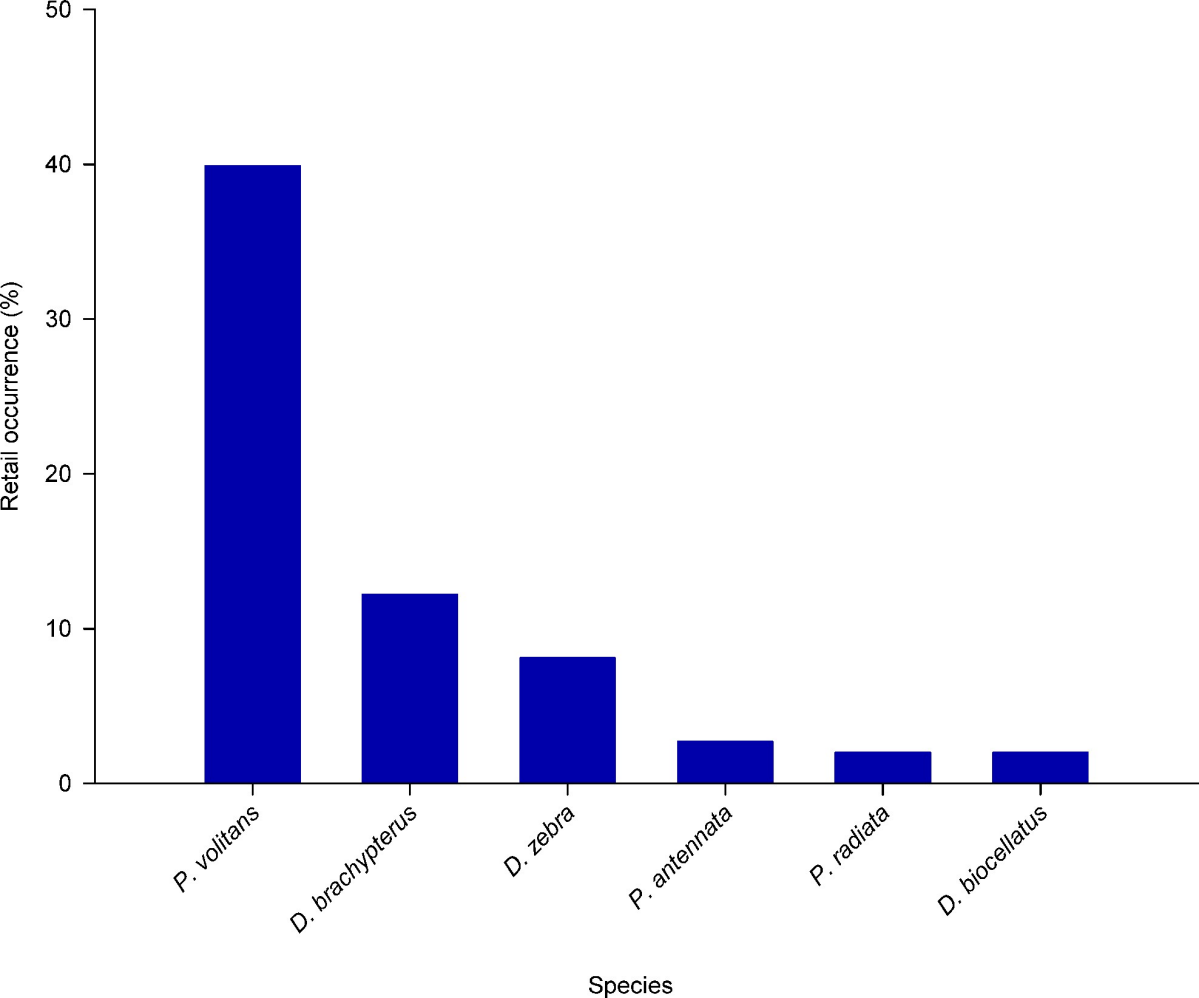
Percent occurrence of six species of lionfish in U.S. retail aquarium stores from 10 coastal states. Surveys were conducted during a two-week period from June 29-July 12, 2017. *P*. *volitans* = 39.9%; *D*. *brachypterus* = 12.2%; *D*. *zebra* = 8.1%; *P*. *antennata* = 2.7%; *P*. *radiata* = 2.0%; *D*. *biocellatus* = 2.0%. No other lionfish species were reported during retail surveys.

We found that 75 of 168 (43.5%) retail shops had at least one species of lionfish in stock (Fig 6). Another 44.6% did not have any lionfish on-site, 8.3% did not answer the store telephone after three contact attempts or had a telephone that was no longer in service, and 3.6% did not participate in the survey. Only six species of lionfishes appeared in surveyed stores. *P*. *volitans* was present in 39.9% of surveyed stores, whereas *Dendrochirus brachypterus* was present in 12.2%, *D*. *zebra* in 8.1%, *Pterois antennata* in 2.7%, *Pterois radiata* in 2.0%, and *Dendrochirus biocellatus* in 2.0% of retail shops (Fig 6).

## Discussion

We identified considerable variation in species diversity and volume at both the import and retail level. The marine ornamental trade is a potentially strong introduction pathway for two species of lionfishes, but moderate to very weak for others. Lionfish import is highly concentrated at the port of Los Angeles, and most specimens originate from the Philippines and Indonesia. Retail surveys indicated a much more limited diversity of lionfishes than previously thought, especially when compared to stock lists provided by online vendors. The genera *Ebosia* and *Brachypterois* were entirely absent from trade at both the import and retail level. Ultimately, these data indicate that there is substantial variation in the volume, diversity, and destination of lionfishes in trade, suggesting that risk is not uniformly distributed across the subfamily.

Marine introductions originating from the aquarium trade are historically rare but are increasingly documented [40]. In Florida (USA) alone, at least 36 marine non-native fishes have been introduced by deliberate or unintentional release across various pathways [41], with many of these introductions occurring through hobbyist release [28]. For example, the panther grouper *Chromileptes altivelis* has been reported from several locations in the Atlantic and Gulf of Mexico, but is not thought to have established [42]. While few introductions originating from the marine aquarium trade have resulted in establishment and spread, two notable exceptions have had major consequences. First, the State of California (USA) has spent considerable time and resources to eradicate and prevent future establishment of the marine algae *Caulerpa taxifolia* [43]. Second, the spread of the invasive lionfish complex in the Atlantic Ocean, Gulf of Mexico, and Greater Caribbean has led to reductions in native species abundance, diversity, and recruitment success [13,15]. While it is not especially common, some marine fishes have established and spread through alternative pathways. The regal demoiselle *Neopomacentrus cyanomos* has established populations in the Gulf of Mexico by traveling within structure underneath petroleum platforms, though the impacts of its spread are currently unknown [44].

The relative volume of lionfish trade in the U.S. is low compared to many other taxa. From 2004 to 2005, over 11 million marine fishes were imported [8], suggesting that lionfishes represent less than 0.5% of total imports by volume. However, more than half of that trade was comprised of just 20 species [8]. In some cases, a single species can account for nearly 10% of total import volume [8]. Despite high trade volumes, none of these species have established populations outside of their native ranges. The lack of establishment success of many high-volume species highlights the importance of species characteristics in predicting risk, and the utility of characterizing the trade of species that share common features with known invaders.

The diversity of lionfishes available directly to hobbyists is likely much lower than previously thought. Many online retailers advertise and presumably sell a diverse stock list of Pteroinae directly to the public, but that diversity was not apparent in our retail survey results. For California, Williams et al. [37] reported the online availability of 12 species to hobbyists from internet sources. At the retail level, our survey found only six species available in 10 coastal states, only five of which were found in 50 Californian retail stores. Additionally, the presence or absence of lionfishes in our survey was verified with current stock lists, whereas previous surveys only reported the diversity of lionfishes advertised online [37,45]. Lower species diversity at the retail level is also supported by the LEMIS and Hawaiian DAR databases which indicate the import of only 11 species of lionfishes, five of which were reported in exceedingly low volumes. Retail surveys were conducted during a two-week period with a representative sample size and therefore the retail trade was not captured in its entirety. Nevertheless, availability of most lionfish species did not vary greatly on a temporal basis and thus our survey likely reflected general availability to hobbyists.

Mortality during wholesaler consolidation and during transit from wholesaler to retailer can vary considerably [30] and may be higher for some species of lionfishes [35]. Collection methods, holding conditions, and shipping practices influence mortality rates across a range of marine taxa [7,29], which may have important implications for the diversity of species available to consumers. The use of chemical anesthetics during collection can have a major impact on the overall survival of specimens in trade [46]. Availability to the hobbyist with ultimately be influenced by this chain of events, and therefore trade volume at the end of the pathway will be reduced. For example, despite a much higher import volume of *D*. *zebra*, our results show that *D*. *brachypterus* is more often encountered in retail settings. This might indicate that *D*. *brachypterus* is more resilient to handling and transport, which affects how likely this species will be encountered by the consumer.

Although import volume, coupled with collection records from Florida and Hawaii, is a direct measure of the total volume of lionfish in the US trade, other factors may influence true propagule pressure. With rare exceptions, hobbyists would have to release the lionfish [31] for there to be propagule pressure into the environment. Maximum species size is an important factors affecting the probability of hobbyist release, which is commonly known as the “tankbuster” effect [2]. There are considerable differences in maximum body size among the lionfishes. The largest species of traded lionfishes *P*. *volitans* reaches 45.0 cm, over three times the size of the smallest traded lionfish *D*. *biocellatus*. Members of *Pterois* are on average larger than members of *Dendrochirus*. Many additional factors may act as modifiers on propagule pressure [39], including species aggression [38], difficulty of care, a perceived danger to oneself or a family member (e.g., venomous fishes), and economic distress or an inability to provide adequate housing. For reptiles and amphibians in a similar US pet trade, high trade volume, large adult size, longevity, and lower retail values were associated with increased incidence of release into the environment [47].

One drawback of using import data as a surrogate for propagule pressure is that it does not account for redistribution after fishes are received at the port of destination. This is especially important for large countries like the United States, which exhibits variation in suitable habitat and ultimately risk [35–36]. Redistribution of lionfish likely plays an important role during transport from wholesaler facilities to retail locations, affecting availability to the consumer. For example, Los Angeles is a major shipping hub for fishes originating from the Indo-Pacific and several major wholesalers are in proximity to the ports so that incoming shipments of fish can be consolidated and quickly redistributed to retail stores. The proximity of hobbyists to suitable habitat and the transportability of the taxa will ultimately affect propagule pressure and the risk of establishment [48]. For example, habitat nearer to roads or footpaths have a greater number of introduced fishes than those in remote locations [49]. Similarly, thermal tolerance and climatic suitability influence the ability of introduced fishes to establish permanent populations [50]. The broad distribution of many lionfishes in the Indo-Pacific [51], and demonstrated cold-tolerance in some species [35], suggests that many species have the potential to establish in the western Atlantic, Gulf of Mexico, and Caribbean. Nevertheless, a large fraction of imported lionfish will be redistributed to destinations which are located far from suitable marine habitats. Therefore, an assessment of trade at the retail level is useful because it identifies trade volume and spatial distribution at the end user destination, where introduction is most likely to occur [31].

Lionfishes share many morphological characteristics, which may provide opportunity for species misidentification. Sri Lanka and Kenya are not included in the historical native range of *P*. *volitans*, yet the LEMIS database reports that *P*. *volitans* comprises 92.9% of lionfish trade from Sri Lanka and 56.6% from Kenya. This is suggestive of misidentification, where the closely related congeners *P*. *miles* and *P*. *russelii*, species that are native to the Western Pacific and Indian Ocean, are likely exported and traded as *P*. *volitans*. Indeed, *P*. *miles* and *P*. *russelii* were both missing from the LEMIS dataset despite considerable collection and export of lionfish within their native ranges (Table 1). Misidentification of large bodied *Pterois* is noteworthy given genetic evidence of hybridization in *P*. *volitans* [52], where future introductions of *P*. *volitans*, *P*. *miles*, *P*. *russelii*, and *P*. *lunulata* may elevate the risk of hybrid vigor. Additionally, several ports received individuals that were not identified to the species level. Over 40% of the 1,200 individuals received by the port of Miami were not identified to the species level, a potential regulatory enforcement issue. These potential errors in database reporting will ultimately affect the volume and diversity of lionfishes that reach the consumer, which has important implications for the management and traceability of the marine ornamental trade.

**Table 1:** Maximum body size of *Pterois*, *Dendrochirus*, and *Parapterois* present in the U.S. ornamental aquarium trade.

Species	Maximum size (TL)	Reference
*D*. *biocellatus*	13.0	[53]
*D*. barberi*	16.5	[54]
*D*. *brachypterus*	17.0	[55]
*D*. *zebra*	25.0	[56]
*P*. *mombasae*	20.0	[53]
*P*. *antennata*	20.0	[57]
*P*. sphex*	21.0	[54]
*P*. *radiata*	24.0	[55]
*P*. russelii**	30.0	[58]
*P*. miles**	35.0	[58]
*P*. *lunulata*	35.0	[53]
*P*. *volitans*	45.0	[60]
*P*. *heterura*	38.0	[59]

* denotes species reported by the Hawaiian Division of Aquatic Resources.

** denotes species that were not reported in a database, but are likely collected and traded under a species misidentification.

Import data are useful for evaluating the total trade volume of marine fishes entering the United States. Combined with retail surveys to account for modifiers on availability at previous stages in the pathway [7,29–30], managers can better characterize the diversity of species at the end of the pathway where release is most likely to occur [28,31]. By identifying hardy species that occur in high trade volumes, understanding spatial distribution at the import level, and spatial redistribution at the hobbyist level, trade data can focus risk assessment and management towards species with high propagule pressure, a consistent predictor of establishment success [18]. Given previous invasion history in the Pteroinae and a strong pathway for some species, future research should aim to apply proactive risk screening measures such as the Aquatic Species Invasiveness Screening Kit to the subfamily [23] to better inform management.

## Supporting information

S1 Dataset Lionfish imported into the United States between April 2, 2016 and April 1, 2017, reported by the U.S. Fish and Wildlife Service Law Enforcement Management Information System. (XLSX)

S2 Dataset Collection of live lionfish in florida, reported by the Florida Fish and Wildlife Conservation Commission Fish and Wildlife Research Institute’s Annual Commercial Fishery Landings database. (XLSX)

S3 Dataset Occurrence of lionfishes in 168 retail aquarium stores in ten coastal states from June 29^th^ to July 12^th^, 2017. (XLSX)
